# Dietary Intake and Adherence to the Recommendations for Healthy Eating in Patients With Type 1 Diabetes: A Narrative Review

**DOI:** 10.3389/fnut.2021.782670

**Published:** 2021-12-16

**Authors:** Rouzha Pancheva, Desislava Zhelyazkova, Fatme Ahmed, Michal Gillon-Keren, Nataliya Usheva, Yana Bocheva, Mila Boyadzhieva, Georgi Valchev, Yoto Yotov, Violeta Iotova

**Affiliations:** ^1^Department of Hygiene and Epidemiology, Medical University of Varna, Varna, Bulgaria; ^2^Institute of Endocrinology and Diabetes, Schneider Children's Medical Center of Israel, Petah Tikva, Israel; ^3^Department of Social Medicine and Health Care Organization, Medical University of Varna, Varna, Bulgaria; ^4^Department of Clinical Laboratory, Medical University of Varna, Varna, Bulgaria; ^5^Department of Internal Diseases II, Medical University of Varna, Varna, Bulgaria; ^6^Department of Imaging Diagnostics, Interventional Radiology and Radiotherapy, Medical University of Varna, Varna, Bulgaria; ^7^Department of Internal Diseases I, Medical University of Varna, Varna, Bulgaria; ^8^Department of Paediatrics, Medical University of Varna, Varna, Bulgaria

**Keywords:** type 1 diabetes (T1D), nutrition, eating pattern, dietary intake, nutritional behavior, nutritional care

## Abstract

**Background:** Medical nutrition therapy is essential for all people with diabetes, of any type or severity. Compliance with the recommended nutrition is an integral part of the treatment of type 1 diabetes (T1D). It remains unclear to what extent the dietary intake of patients with type 1 diabetes adheres to the recommendations for healthy eating.

**Objective:** The primary aim of our study is to collect and analyze published articles on the nutrition of T1D patients in comparison with the general population and recommendations.

**Research Strategy and Methods:** A literature search for articles, published between January 2006 and July 2021 was conducted, using electronic databases (PubMed and Google Scholar) for all available publications in English and Bulgarian. The process of study selection, identification, screening, eligibility and inclusion followed the PRISMA (Preferred Reporting Items for Systematic Reviews and Meta-Analyses) recommendations for a flowchart. Based on the keywords search, 425 titles were retrieved, of which 27 were selected based on title and abstract. All papers were crosschecked and reviewed for selection by 3 independent reviewers. As a result, 19 titles were eligible and met inclusion criteria for a full review.

**Results:** Energy intake tends to be lower in T1D patients or comparable to controls and in most cases within the general recommendations. The percentage of calories from protein is within the recommendations for children, adolescents and adults. Only two studies showed that T1D patients consume significantly less than the recommendation for total fat intake (<35E%). The median intake of carbohydrates is in the lower end of the recommended 45 to 60E%. The median intake of dietary fiber adjusted for total energy is too low for T1D patients and the general population.

**Conclusion:** Study findings suggested a lack of knowledge or misunderstanding of diabetes dietary management. Patients with T1D, who are being consulted with a dietician as a part of their treatment plan may have better compliance to their recommended diet and as a result, are likely to have better health outcomes. Nutritional therapy should focus not only on glycemic control and pure carbohydrate counting but also on healthy eating and complication prevention.

## Introduction

Type 1 diabetes (T1D) is a chronic autoimmune condition in which the pancreas produces less insulin than required (1). This leads to the inability of the organism to utilize glucose and eventually to hyperglycemia (2). The goal of treatment in T1D is to provide insulin in as physiologic a manner as possible (3). Eating behaviors, dietary control, and physical activity are perceived as an essential parts of the strategies for preventing diabetes-related complications. Therefore, a holistic approach toward the management of T1D is required. Medical nutrition therapy is essential to all people with diabetes, of whatever type or severity (4). Nutrition therapy is recommended for all children, adolescents, and adults with diabetes. Avoidance of deviation in the recommended nutrition is an integral part of the treatment and self-management of T1D. Patients with type 1 diabetes (T1D) are advised to have a healthy lifestyle and maintain adequate body weight. An important role in the improvement of the diet quality and optimizing glycemic control have the meal-time routine and restriction of snacking. Nutritional guidelines are established on the principles of healthy eating (5). Their purpose is to improve glycemic control, prevent acute and chronic diabetic complications, and lower cardiovascular risk. The cultural, ethnic, and family traditions, as well as the cognitive and psychosocial circumstances of the patient, should be taken into consideration when nutritional guidance is given (5). Due to the complexity of diabetes treatment in general, and nutritional therapy in particular, usually, the dietary guidance focuses on adjusting insulin to meals according to their carbohydrate content, and the healthy diet guidance is limited. Hence, as part of the treatment, a customized meal plan should be prepositioned, taking into consideration the individual's features and attempting to achieve the best-balanced diet possible for the specific patient. Overall, it remains unclear to what extent the dietary intake of patients with type 1 diabetes adheres to the recommendations for healthy eating and if the nutrition of this vulnerable group of patients is healthier compared to the general population. There is a need to synthesize the emerging research and evaluate important distinctions to further guide nutrition education.

## Aim

Our study aims to collect and analyze published articles on the nutrition of T1D patients in comparison with the general population and recommendations.

## Methods

### Search Strategy

**Figure 1:**
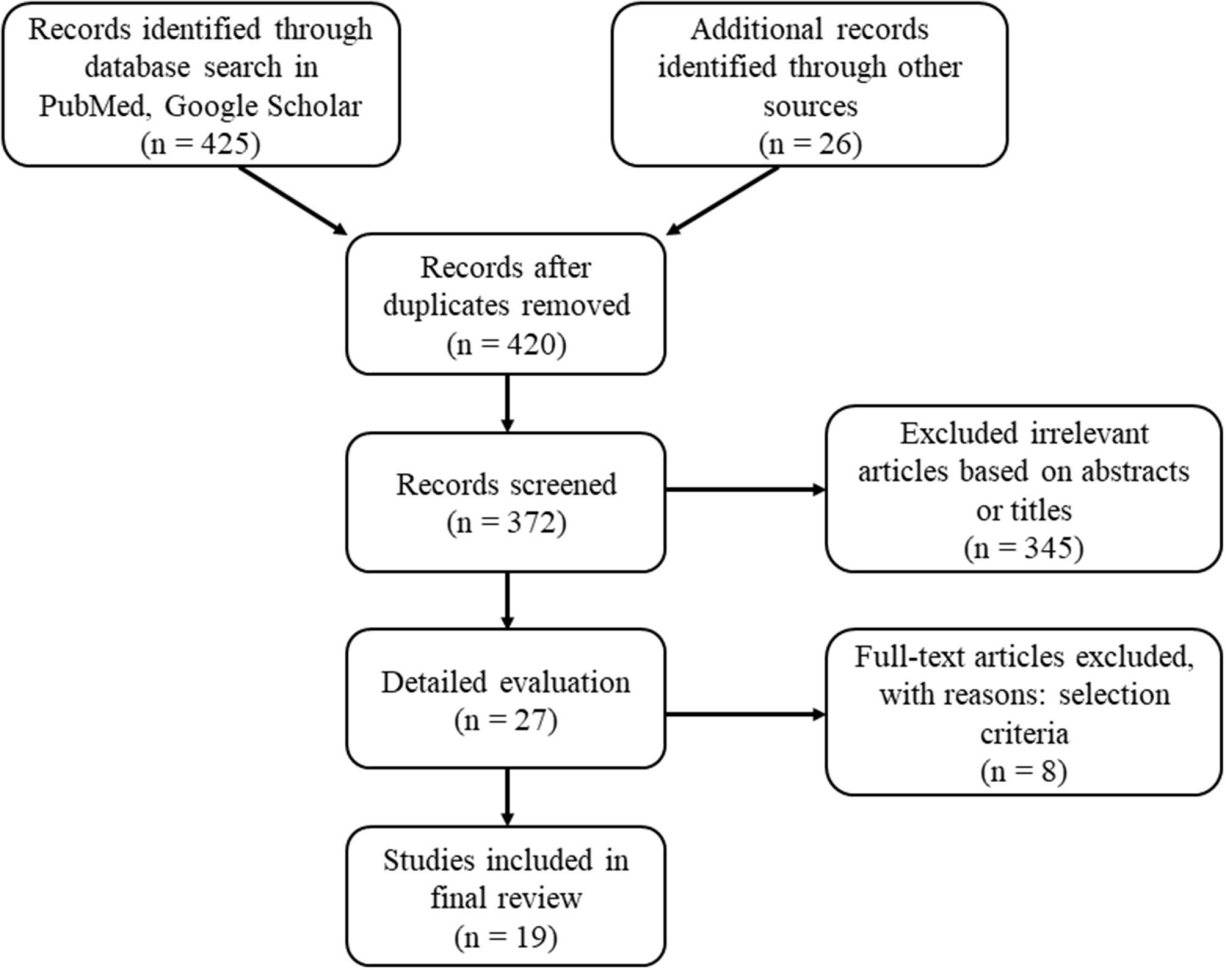
Flow chart of the included studies regarding dietary intake among adult patients with T1D.

A literature search among articles, published between January 2006 and July 2021 was conducted, which is a 15-year period- the most recent, that encompasses enough articles to see the relevant trend in nutrition, using electronic databases (PubMed and Google Scholar) for all available publications in English and Bulgarian. Keywords included “type 1 diabetes,” “nutrition,” “diet,” “dietary habits,” “nutritional guidelines,” “nutritional recommendations,” “dietary regime,” “nutritional plan,” “dietary plan,” “eating plan,” “diet plan,” “meal plan,” “nutritional behavior,” “nutritional care,” “eating pattern,” “dietary intake,” “macronutrients,” “micronutrients.” Synonyms were used to increase search sensitivity. References in key papers were also explored. The process of study selection, identification, screening, eligibility and inclusion followed the PRISMA recommendations for a flowchart (Figure 1). Based on the keywords search, 425 titles were retrieved, of which 27 were selected based on title and abstract. All papers were crosschecked and reviewed for selection by three independent reviewers. As a result, 18 titles were eligible, and met inclusion criteria for a full review.

### Selection Criteria

The inclusion criteria were as follows: 1/ focused on nutrition or dietary habits of T1D patients, 2/ included information on dietary intakes—in particular energy, nutrients, food groups, 3/ reflected adherence to dietary recommendations, 4/ compared intake in patients with T1D with general population groups. Studies were excluded if they: 1/ focused on nutrition interventions, 2/ explored the role of a dietitian, 3/ reported metabolic or cardiovascular complications of medical nutrition therapy, 4/ discussed nutrition-related mortality, 5/ analyzed knowledge and beliefs, 6/ commented on the effect of alternative eating patterns such as low carbohydrate diet, veganism, etc., 7/ analyzed data only from low- income countries, 8/ were randomized control trials (RCTs) or reviews.

## Data Extraction

Data extraction was performed by three reviewers using a predefined data extraction table. The following information was included: (1) First author and year of publication (2) Sample size (3) Age group (4) Main measures studied (5) Study design (6) Diet assessment method (7) Main results: energy and nutrient intake and/or frequency of consumption of food groups. Any discrepancies in data extraction were discussed and resolved by consensus among the reviewers. The main characteristics of the selected studies are presented in Table 1.

**Table 1:** Main measures and nutrition outcomes among T1D patients.

**References**	**Number of T1D patients**	**Age group**	**Main measures studied**	**Comparison with**	**Method of dietary assessment**	**Main results**
Moore (6)	55	2–7 y.	Macronutrients, food groups	Recommendations	3-day dietary record	Carbohydrates 48.20 ± 0.09E%, protein 14.51 ± 0.04E%, fat 33.42 ± 0.07E% (within recommendations of ADA), saturated fat 13.07 ± 3.39E%; Subjects are closest to the recommendation for fruit, dairy and protein; 43% of patients meet the recommendation for vegetables; 89% do not meet the recommendation for substitution of half of their dietary grains with whole grains.
Seckel et al. (7)	24	<7 y.	Macronutrients, food groups	Recommendations	3-day weighed food diaries	Carbohydrates 48 ± 4E%, protein 16 ± 2E%, fat 33 ± 5E%, saturated fat 15 ± 3E%; Just over half of the children meet the Australian recommended number of servings for their age and gender-−61% of the subjects meet the recommendation for fruit, 61% for dairy, 56% for bread and cereals; No child meets the recommended servings of vegetables.
Mehta et al. (8)	67	2–12 y.	Macronutrients, food groups	Control group (NHANES), Recommendations	Multiple 24-h dietary recalls	Greater proportions of children with T1D meet daily recommendations for vegetables-−22 vs. 13% for the controls, whole grains 12 vs. 5%, dairy 55 vs. 36%; Similar proportions meet daily fruit recommendations 40 vs. 33%; Children with T1D consume more saturated fat-−14E% vs. 12E% for the controls; Fiber intake is very low in both groups; 1/3 of both groups limits total fat to recommended levels.
Maffei et al. (9)	229	6–16 y.	Energy, macronutrients	Comparison between 2 time periods: group A- 2009 and group B- 2019	7-day-diet history	Energy intake is not significantly different in the two groups (group A vs. group B): 1,765 ± 282 kcal/day vs. 1,820 ± 295 kcal/day; Carbohydrate intake is significantly lower in group B: 53.8 ± 5.1?% vs. 48.3 ± 5.2?%. Protein intake 14.9 ± 2.1?% vs. 17.5 ± 4.3?% and fat intake 31.0 ± 5.3?% vs. 33.9 ± 5.3?% is significantly higher in group B.
Papadaki et al. (10)	41	6–17 y.	Macronutrients, food groups	Control group, Recommendations	24-h recall	Carbohydrate intake is lower than the recommended: 44E% vs. 45E%, recommendation: >55E%; Protein intake is within the recommended: 17E% vs. 14.6E%, recommendation: 12–20E%; Patients and controls exceed the recommendations for total fat: 40E%, vs. 41E%, recommendation: <35E% and for saturated fat: 13E% vs. 14E%, recommendation: <10 E%; Dietary fibred: 19 vs. 15g/day, recommendation:.>20 g/day; Participants with T1D consume more dairy products, vegetables and fruits, and less meats and cereals.
Dłuzniak-Gołaska et al. (11)	194	8–18 y.	Dietary pattern	Recommendations	FFQ	The average healthy diet score obtained by all the patients is only 27.6 ± 11.1 and ranged from 3.8 to 61.0 (maximum value of 100).
Overby et al. (12)	177	9–13 y.	Energy, macronutrients, food groups	Control group	Standardized 4-day food records	Protein intake is within the recommendation; There are no differences in energy and protein intake between T1D patients and controls; Total fat 33–35E%, and saturated fat 14–15E%, is higher than the recommended; Fiber intake 16–19 g/day, fruits and vegetables intake 210g/day, are lower than the recommendation.
Helgeson et al. (13)	132	10–14 y.	Energy, macronutrients	Control group	3 times 24-h recall	Energy consumption was below recommendations. In both groups, the percentage of calories from carbohydrates and protein are within recommendations; Participants with T1D exceed the recommended fat intake, their diet consists of a greater percentage of protein, total fat, a smaller percentage of carbohydrates and less sugar, compared to controls.
Baechle et al. (14)	712	11–19 y.	Carbohydrates, meal frequency	Control group	FFQ	A total daily carbohydrate intake of 75.9 g is lower in T1D compared to controls. Carbohydrate intake is consistently lower at all eating occasions compared to controls. Participants with T1D consumed breakfast, lunch, dinner, and snacks more frequently.
Mackey et al. (15)	257	12 ± 1.2 y.	Macronutrients	Recommendations	2 times 24-h recall	Lower intake of carbohydrates 24.7–26.6E%, recommendation: 50–55E%; Higher intake of protein 44.7–48.8E%, recommendation: 15–20E%; Higher intake of fats 35.6–36.1E%, recommendation <35E%; 47.6% meet the minimum recommendation for dietary fiber.
Faulkner et al. (16)	64	13–18 y.	Energy, macronutrients	Control group	24-h recall	Energy consumption within recommendations, with no significant differences between T1D and controls. Significantly lower consumption of carbohydrates in T1D compared to controls: 296 vs. 411 g/day; Similar protein intake: 99 vs. 94 g/day; Higher total fat 115 vs. 90 g/day; Higher saturated fat intake 40 vs. 30 g/day.
Lodefalk et al. (17)	174	13–19 y.	Macronutrient, food groups	Control group, recommendations, food groups	FFQ, 4-day food record	Energy intake within Swedish recommendations. The intake of protein is higher than recommended in boys, but not in girls; The intake of saturated fat is higher than recommended in both boys and girls; The intake of polyunsaturated fat is lower than Swedish recommendations in both boys and girls; The intake of fiber in girls is lower than the calculated recommendation; Both male and female patients consume more protein, less sucrose and more fiber than healthy Swedish adolescents and young adults; Patients eat more regularly, and more often fruit and fruit juice, potatoes and root vegetables, meat, fish, egg, offal and sugar-free sweets.
Ewers et al. (18)	774	>18 y.	Macronutrient, food groups	Control group, recommendations	FFQ	Energy intake in both groups was lower compared with the general population. Adherence to dietary recommendations for fiber, saturated fat, vegetables, fruit and fish are low in all groups, but lowest in the general population.
Snell-Bergeon et al. (19)	571	19–56 y.	Macronutrients	Control group, recommendations	FFQ	Adults with T1D report a diet higher in fat, saturated fat and protein, but lower in carbohydrates; Fewer than half of those with type 1 diabetes meet dietary guidelines for fat and carbohydrate intake; Only 16% restrict saturated fat to <10% of daily energy intake; Adults with type 1 diabetes are significantly less likely to meet dietary guidelines than controls.
Soedamah et al. (20)	1,102	33 ± 10 y.	Energy, macronutrients	Recommendations	3-day dietary record	Generally, the reported energy intake is lower. European recommendations for adequate nutrient intakes are followed by individuals with type 1 diabetes for protein: 78% at follow-up, moderately for fat 40%, carbohydrate 41%, and cholesterol 47%, but poorly for fiber 2.4%, and saturated fat 13%.
Ahola et al. (21)	817	>35 y.	Energy, macronutrients, micronutrients	Recommendations	2 times 3-day food record	Diet low in carbohydrates and fiber, but high in fat; Only 28% restrict saturated fatty acid to <10% of their daily energy intake; One-fourth of the patients report higher than recommended sucrose intake; Salt recommendations are frequently exceeded; Of the micronutrients, the recommendations for vitamin A, vitamin D, folate, and iron are mostly frequently unmet.
Giorgioni et al. (22)	60	35.8 ± 11.3 y.	Micronutrients	Recommendations	7-day food records	Good adherence to recommendations for vitamins A, B_6_, B_12_, and C; Intermediate adherence to folate, vitamin E, vitamin D; Low adherence to potassium, zinc, copper, magnesium, calcium; Low adherence to iron in women.
Jaakset al. (23)	100	41.7 ± 16.3 y.	Macronutrients, food groups	Within the group, recommendations	24-h recall,	Generally low carbohydrate, normal protein, high fat, low fiber diet; No consistent differences in dietary intake across subgroups of patients, who are having additional nutrition education.
Usheva et al. (24)	118	42.6 ± 10.5y.	Dietary habits, food groups, consumption	Control group	FFQ	Significantly better dietary habits regarding the everyday presence of breakfast, lunch, dinner, number of portions for fruits; Significantly more often junk foods; Higher number of glasses of sweetened beverages.

## Results

### Energy Intake

Energy intake in T1D patients tends to be lower or similar to controls (13, 16) and in most cases within the general recommendations (17). Differences in energy intake between T1D patients and T2D were demonstrated in a Finish study from 2006 (16). Dietary energy was measured in three groups—T1D, T2D, and controls. The three groups did not differ significantly. Similar to that in a more recent study by Ewers et al. the median daily energy intake was lower in patients with T1D and even after adjustments for age, sex, BMI, physical activity, and education level remained lower in T1D (−9.9%; 95% CI, −11.2 to −8.6; *P* < 0.001) compared to the general population. In these studies, limitations were discussed, such as general underreporting, which is frequently demonstrated in both groups (18). Plausible energy consumption in 89% of T1D patients was shown also in another big study—EURODIAB Prospective complications study (20) which showed that even after a 7 year follow-up, the energy consumption stayed the same.

### Protein

The percentage of calories from protein is within recommendations for children, adolescents, and adults in most of the studies (13, 15). Only three of the articles report a higher protein intake in patients with T1D. In some longitudinal cohort studies following patients for 7–10 years, there is a difference in the reported protein intake over time. In one study—a moderate decrease with time within the recommended intake was shown (20) and in another—increased intake over 10 years (9).

### Fat and Fatty Acids

Only two studies show that T1D patients consume significantly less than the recommendation for total fat intake (<35E%) (18). In more than 70% of studies, <50% of included patients meet dietary guidelines for fat intake. However, even in such cases, the mean intake of saturated fatty acids exceeds the national recommendation, as only <1/3 follow the guidelines for restriction of saturated fatty acids to <10% of the daily energy intake. In addition, in a large study, children with T1D consumed more saturated fat than the NHANES (National Health and Nutrition Examination Survey) children (14 vs. 12% total energy intake, *p* = 0.0009) (8).

### Carbohydrate

Adherence to carbohydrate recommendations is the most studied aspect of nutrition studies of patients with T1D. A study published in 2012 revealed that among 187 adult patients with T1D in Finland, 51% consumed 45.1 ± 6.6% of energy from carbohydrates (5), which is lower than the recommended range. This observation was supported by another cross-sectional study among adult patients with T1D and T2D in Denmark, published in 2018. It was found that patients consumed fewer carbohydrates than the healthy controls. Moreover, they had a 20–50% lower intake of added sugar than the healthy participants. Compared to the general population, those with T1D were closer to fulfilling the recommendations for reducing the intake of added sugar (97 vs. 67%). Their median intake of carbohydrates was about 45E%, which makes it in the lower end of the recommended 45–60E% (18).

### Fiber

The soluble fiber in vegetables, legumes and fruit may be particularly useful in helping to reduce lipid levels and postpone metabolic complications (25). Dietary surveys show that the median intake of dietary fiber adjusted for total energy is higher in patients with diabetes in comparison to the general population (29.31 vs. 23 g/10 MJ), still, the fiber intake is too low for both groups (18). A similar trend is supported by other studies from 2006 and 2008 (10, 12, 13). Compared to national recommendations in a more recent cross-sectional study with adult patients, it was found that the fiber intake was lower than the recommended with no difference between patients and the general population (8.2 ± 0.1 vs. 7.4 ± 0.5 g/1,000 kcal) (26).

### Micronutrients

In a few studies in this field, the micronutrients intake among patients with type 1 diabetes was lower than recommended. Most of the patients didn't follow the vitamin D and folate intake guidelines. Two-thirds complied with diet, with a sufficient amount of vitamin A. Most (82%) of the patients met the calcium intake recommendation, while only a half reported a diet with adequate iron intake (5, 21, 22).

### Fruits and Vegetables

Studies focused on deviation in nutrition examined how patients balanced their intake of fruits and vegetables with their blood glucose and insulin levels. Those with T1D consumed more vegetables (6, 7, 18) than healthy controls and less than the nutrition recommendation (12, 18). Children <7 years of age were also prone to eat fewer vegetables than the recommended (7). In two studies, adults with type 1 diabetes reported a higher intake of vegetables (up to 20%) than healthy patients (17, 18).

### Other Aspects of the Diet

Regarding other foods and nutrients with long-term health effects, such as salt and alcohol—only 27% of the examined didn't exceed the recommended salt intake. In comparison to men, women met more frequently the salt guidelines (14 vs. 36%; *P* 0.001) (5). Compared to the general population, the patients with T1D reported a 30% lower intake of added sugar and 20–50% lower intake of alcohol, and the difference reached statistical significance. However, the participants with T1D demonstrate a 37% higher alcohol consumption in comparison to the ones with T2D (*P* < 0.001) (18). The mean alcohol consumption was 2.9 g/day, with a range between 0 and 119 g/day. On average, alcohol intake provided 1 E%. Most (82%) of the participants complied with the recommended level of not exceeding 5 E% (5).

## Discussion and Conclusion

Adherence to healthy diet recommendations for daily energy intake and range of intake of macronutrients are essential in the management of diabetes and prevention of diabetes-related complications. Compared to the general population, patients with diabetes have better nutritional education (18). The dietary consumption of patients with type 1 diabetes largely differs from the general population, which is due to mainly high fat (2, 9, 15, 17, 19, 27) and low carbohydrate consumption (7, 9, 10, 14–16, 20, 28). According to a smaller part of the studies, carbohydrate (13) is within recommendations in a much lower percentage of patients. Protein intake is mostly within age recommended for percent of energy (10, 12, 15, 16, 19).

Energy intake varies greatly among subjects daily due to differences in age, growth rate, physical activity, and essential environmental factors such as availability of food (29). It should be sufficient to maintain optimal body weight. The energy needs are increased in T1D during insulin deprivation (26) due to catabolic processes. When insulin therapy is introduced, energy intake corresponds to the one in a healthy state, to maintain good glycemic control. The significance of energy intake in T1D patients was shown in a study by Takase et al. in 2019 (27). A large number of studies have provided evidence that energy intake had effects on blood glucose levels (30). There is a strong positive correlation between energy consumption and visceral fat, which is a risk factor for cardiovascular complications in the general population. Sometimes excess energy intake can be due to poor insulin adjustment, causing recurrent episodes of hypoglycemia, and also as a result of eating snacks due to a particular insulin regimen (e.g., Humulin R or Actrapid). Excessive energy intake can lead to weight gain and related complications, such as insulin resistance and cardiovascular diseases (CVD).

Fat is one of the main macronutrients with a metabolic effect which has an immediate effect on glycemia (29). High-fat meals initially reduce the glycemic excursion for up to 90 min after the meal. This is most likely due to the effect of fat in delaying gastric emptying. Late sustained hyperglycemia is noted when meals high in protein and fat are eaten. That is why there is a need for an evidence-based, safe, and practical method to guide insulin adjustments for high-fat, high-protein meals (31). In the longer term, a diet rich in fat and particularly saturated fat could raise cholesterol, especially LDL-cholesterol which is linked to CVD in diabetic patients. Protein is important for growth, especially in childhood, and for the maintenance of muscle mass in older age. Protein metabolism is significantly affected during insulin deprivation. A greater increase in whole-body protein breakdown than protein synthesis occurs resulting in a net protein loss (26). Protein requires insulin for metabolism, as do carbohydrates and fat, but has minimal effects on blood glucose levels (32). It was found that protein and fats have a major impact on post-prandial blood glucose levels (33). A study from 2013, demonstrated an effect of dietary protein independent of fat on post-prandial glycemia in children with T1D. Importantly, the glycemic rise after protein consumption was shown in meals of both high fat and low-fat contents, with identical carbohydrate quantities (29). The possible mechanism is that protein may lead to delayed hyperglycemia by gluconeogenesis and increased glucagon secretion (34). In the present review, according to eight of the examined studies, there is no significant difference between the protein intake of patients and healthy controls. Moreover, both groups are under the upper limit of the recommendation for protein intake. Only three of the studies reported high protein intake for the patients. Furthermore, two of them found a higher protein intake in diabetes patients in comparison to healthy controls (5, 35).

On the other hand, carbohydrate intake was lower compared to the general population. Good glycemic control is important to reduce the risk of complications. People at risk are advised to avoid simple carbohydrates to control glycemia and reduce the risk of cardiovascular disease. Close adherence to carbohydrate intake recommendations is associated with better glycemic control. The disparity between carbohydrate intake and insulin can result in long-term complications from hypo- and hyperglycemia. Patients have been mostly educated to calculate carbohydrate units and to adjust their insulin dose to the carbohydrate intake accounting for their blood glucose level. Thus, their awareness about this macronutrient is highest (36). Limiting carbohydrate intake leads to increased fat intake. It has been speculated that dietary management of diabetes focuses mainly on carbohydrate intake and that fat-containing foods may be appealing to avoid blood sugar fluctuations, and because exogenous insulin seems not to be needed for carbohydrate-free, potentially high-fat foods (37). On the other hand, the type of fat is more important than the amount of fat. Therefore, higher consumption of monounsaturated and omega-3 fatty acids should be encouraged instead of saturated fat, to improve blood lipid profile and reduce the risk of CVD. The general trend of underconsumption of carbohydrates may also be due to the focus on carbohydrate counting in the nutritional education of patients and to the visibility of blood glucose level fluctuation with the increasing use of continuous glucose measurement (CGM) devices.

In T1D patients, fiber can slow the absorption of sugar and help control blood glucose levels. Increasing the amount of fiber in the diet can help manage diabetes. A review by Reynolds and colleagues in 2020 demonstrated that for people with type 1 diabetes rise in the consumption of fiber from 19 to 35 g is an important component of their diet (38). There was a clear dose-response relationship resulting in improvements in glycemic control, blood lipids, body weight, and inflammation, as well as a reduction in premature mortality.

These benefits were not confined to any fiber type or any type of diabetes and were apparent across the range of intakes. Though greater improvements in glycemic control were observed for those moving from low to moderate and high intakes. While more studies are needed, the results from this review demonstrate lower consumption of fiber (8), compared to recommendations and comparable or higher than controls.

Due to the high level of oxidative stress and inflammation in the blood vessels, there may be a greater need for micronutrients for patients with T1D. Despite this rationale, the recommendations of the American Diabetes Association (ADA) and the International Society for Pediatric and Adolescent Diabetes (ISPAD) are for consumption similar to that of the general population. This is an important area for research because it is key to identifying risk factors and deviations in nutrition (5, 18).

Comparing dietary quality, the studies found that vegetables and fruit intake were below recommendations in persons with diabetes compared to the general population. It can be assumed that consumption is low due to the concern of a higher post-prandial glucose level. Moreover, a large proportion of patients reported a diet with high salt and low sugar intake. Comparing diets in healthy controls and participants with type 1 diabetes, the latter seemed to be slightly better. This could be explained by the fact that patients are continuously educated to follow a structured healthy diet and have a few deviations in their nutrition.

According to the study of Sajjadpour et al. adults with type 1 diabetes were better educated and healthier than the controls (39). The conclusion was that dietary patterns rich in vegetables and fruits may be inversely associated with dyslipidemia in patients with T1DM and that such results if supported by other studies, can be used for developing interventions that aim to promote healthy eating for the prevention of cardiovascular diseases in these patients. The significance of fruits and vegetables in the diet of patients with T1D was outlined in the 2021 study in Iran with 179 women with T1D (39). Three major dietary patterns were found: one with a predominance of grain, legume, and nut, second mostly with fruits and vegetables, and third with high consumption of high-calorie foods, salty snacks, sweets, and desserts. After adjustment for age, body mass index, and energy intake, subjects who were eating more fruits and vegetables had significantly lower levels of LDL (*p* = 0.01), triglyceride (*p* = 0.02), and total cholesterol (*p* = 0.01).

A large number of studies have provided evidence for the correlation between alcohol consumption and glycemic control (40, 41) proving that alcohol consumption is an additional disadvantage in the long-term therapy of T1D patients.

Still, the complex assessment of diet is a difficult task, due to which fundamental indicators for healthy nutrition have been developed. Assessment of diet quality as a whole is essential. Some of the diet quality indices include; the Dietary Approaches to Stop Hypertension (DASH) index, the Healthy Eating Index 2015 (HEI-2015), and a modified Mediterranean Diet Quality Index (mKIDMED) (42). These have been applied in nutritional studies for patients with T1D.

Dietary tendencies are reflected in the Healthy Eating Index-2005 (HEI-2005) applied to children with T1D and the general population, which shows a higher score for the patients mainly due to lower sugar consumption.

Interestingly, as patients age, the consumption of carbohydrates decreases, and the fat and protein intake increases. When patients are young and their parents play an important role in their nutrition, the adherence to the recommendations is higher. However, patient compliance diminishes as they get older. These findings support the hypothesis that patients with T1D need continued nutrition education to build a base of a healthy lifestyle. Study findings suggested a lack of knowledge or misunderstanding of diabetes dietary management. Patients with T1D, who are being consulted with a dietician as part of their treatment plan may have better compliance to their recommended diet and as a result, are likely to have better health outcomes in the longer term. Patients with diabetes are at increased risk of CVD, therefore they must receive dietary guidance. Nutritional therapy should focus not only on glycemic control and pure carbohydrate counting but also on healthy eating and complication prevention. Hence, the focus should not be on the macronutrient composition of the diet (carbohydrates, fat, and protein) but rather on healthy eating patterns.

The current study has several strengths. First, this is the first recent narrative review of observational studies on dietary intake of T1D patients and compliance with recommendations. Second, it has a focus on comparison with healthy controls based on the most comprehensive literature search to date. Third, all included studies were published in recent years, suggesting the proposed topic of the study is of potential scientific interest. Fourth, our results show a common dietary trend in different countries and time periods. It is essential that these are clearly defined and appropriate action taken. Bringing these similarities to the attention of the scientific community is also of importance for the quality of clinical care for T1D patients *per se*.

The limitations of this narrative review should also be highlighted. Firstly, the studies included in the review are heterogeneous- presenting diverse population groups, with ununiform recommendations for healthy nutrition, and various tools to assess it, which may have specifics related to time periods and may differ from country to country. Secondly, due to the inclusion-exclusion criteria, only 19 studies were identified and recruited in the analysis. These limitations should be taken into consideration while assessing the significance of this study.

## Conclusion

Study findings suggested a lack of knowledge or misunderstanding of diabetes dietary management. Patients with T1D, who are being consulted with a dietician as a part of their treatment plan may have better compliance to the recommended diet and as a result, are likely to have better outcomes. Nutritional therapy should focus not only on glycemic control and pure carbohydrate counting but also on healthy eating patterns and complication prevention.

## Author Contributions

YY and VI conceptualized and supervised the study. RP conceived the idea. FA and DZ selected and retrieved relevant papers. RP, DZ, FA, and MG-K drafted this review. The remaining authors were the guarantors of the overall content. All authors revised and approved the final manuscript.

## Funding

This study was supported by a research grant DN 13/3, from the Scientific Research Fund at the Ministry of Education and Science of Bulgaria.

## Conflict of Interest

The authors declare that the research was conducted in the absence of any commercial or financial relationships that could be construed as a potential conflict of interest.

## Publisher's Note

All claims expressed in this article are solely those of the authors and do not necessarily represent those of their affiliated organizations, or those of the publisher, the editors and the reviewers. Any product that may be evaluated in this article, or claim that may be made by its manufacturer, is not guaranteed or endorsed by the publisher.
